# Blood brain barrier dysfunction and delayed neurological deficits in mild traumatic brain injury induced by blast shock waves

**DOI:** 10.3389/fncel.2014.00232

**Published:** 2014-08-13

**Authors:** Ashok K. Shetty, Vikas Mishra, Maheedhar Kodali, Bharathi Hattiangady

**Affiliations:** ^1^Texas A&M Health Science Center College of Medicine at Scott & White, Institute for Regenerative MedicineTemple, TX, USA; ^2^Department of Molecular and Cellular Medicine, Texas A&M Health Science Center College of MedicineCollege Station, TX, USA; ^3^Research Service, Olin E. Teague Veterans Affairs Medical Center, Central Texas Veterans Health Care SystemTemple, TX, USA

**Keywords:** blast-related brain injury, blast shock waves, blood-brain barrier leakage, chronic traumatic encephalopathy, mild traumatic brain injury, neuroinflammation, oxidative stress, vascular lesions

## Abstract

Mild traumatic brain injury (mTBI) resulting from exposure to blast shock waves (BSWs) is one of the most predominant causes of illnesses among veterans who served in the recent Iraq and Afghanistan wars. Such mTBI can also happen to civilians if exposed to shock waves of bomb attacks by terrorists. While cognitive problems, memory dysfunction, depression, anxiety and diffuse white matter injury have been observed at both early and/or delayed time-points, an initial brain pathology resulting from exposure to BSWs appears to be the dysfunction or disruption of the blood-brain barrier (BBB). Studies in animal models suggest that exposure to relatively milder BSWs (123 kPa) initially induces free radical generating enzymes in and around brain capillaries, which enhances oxidative stress resulting in loss of tight junction (TJ) proteins, edema formation, and leakiness of BBB with disruption or loss of its components pericytes and astrocyte end-feet. On the other hand, exposure to more intense BSWs (145–323 kPa) causes acute disruption of the BBB with vascular lesions in the brain. Both of these scenarios lead to apoptosis of endothelial and neural cells and neuroinflammation in and around capillaries, which may progress into chronic traumatic encephalopathy (CTE) and/or a variety of neurological impairments, depending on brain regions that are afflicted with such lesions. This review discusses studies that examined alterations in the brain milieu causing dysfunction or disruption of the BBB and neuroinflammation following exposure to different intensities of BSWs. Furthermore, potential of early intervention strategies capable of easing oxidative stress, repairing the BBB or blocking inflammation for minimizing delayed neurological deficits resulting from exposure to BSWs is conferred.

## Introduction

Exposure to shock waves stemming from ignition of explosive devices can produce considerable injury to both torso and brain (Rosenfeld et al., 2013; Kovacs et al., 2014). The danger for such exposures is extremely great to military personnel in contemporary warfare but they can also occur to civilians in circumstances such as bomb detonations by terrorists. The use of individual body protection systems by military personnel has diminished blast-related fatal thoracic and abdominal injuries in the recent Operation Iraqi Freedom and Operation Enduring Freedom wars in Afghanistan (Rosenfeld et al., 2013; Kovacs et al., 2014). However, a significant fraction of military personnel exposed to blast shock waves (BSWs) exhibit mild traumatic brain injury (mTBI; Ling and Ecklund, 2011). Persons exposed to BSWs display diverse neurological deficits depending upon the severity of shock waves and the region of brain affected by these shock waves. The symptoms may range from temporary mild cognitive problems to a more serious and continuing brain dysfunction characterized by significant memory and mood impairments, post-traumatic epilepsy or coma (Kovacs et al., 2014). Injuries from blast waves have been categorized as primary, secondary, tertiary and quaternary types (see Kovacs et al., 2014 for more details). Primary injury refers to brain damage happening directly from exposure to the explosive blast wave, secondary injury is brain damage owing to being hit by bomb constituents (e.g., fragments, rocks) driven by blast waves, and tertiary injury is a crash related brain damage resulting from being physically thrown into other objects or the ground. Quaternary injury refers to all other forms of injury ensuing through a blast, which include fireball related burns, exposure to toxic fumes and radiation released at blast sites (Phillips, 1986; Kovacs et al., 2014).

Multiple experimental studies have revealed that blast overpressure results from a sudden discharge of energy that produces rapid expansion of high-pressure gas into the ambient atmosphere. When a pressure pulse of random form cruises through a medium, higher-pressure components of the pulse travel faster than lower pressure parts, which causes the wave components to add gainfully and generate a sudden increase in pressure, or a shock (Cullis, 2001; Yeoh et al., 2013). However, once rarefaction waves catch up to the shock front, the shock begins to degrade into a blast wave. The resulting pressure time wave shape makes a Friedlander curve where pressure falls swiftly after the peak and then transitorily plunges lower than the atmospheric level (Cullis, 2001; Yeoh et al., 2013). The area of the curve denoting greater pressure than atmospheric pressure is called the positive phase whereas the region indicating lower pressure than the atmospheric pressure is called the negative phase (Yeoh et al., 2013).

Several mechanisms have been proposed for the primary brain injury caused by BSWs. A widely accepted hypothesis is that, shock waves traverse the brain tissue causing its acceleration and deformation; the degree of brain damage would depend upon the shape of BSW, its peak overpressure and pulse duration and the tissues’ natural resonant frequencies (Desmoulin and Dionne, 2009; Magnuson et al., 2012; Kovacs et al., 2014). Another supposition is that shock waves first impact the torso, the kinetic energy of these waves gets transferred into hydraulic energy in the cardiovascular system and causes a rapid physical displacement of blood which moves through blood vessels from the high pressure body cavity to the low-pressure cranial cavity, causing damage to tiny cerebral blood vessels and blood-brain barrier (BBB; Chen et al., 2013). Studies in animal models have suggested that both direct and indirect mechanisms (when torso is not protected with a body armor such as Kevlar) contribute to the pathophysiology of blast-related mTBI through dysfunction or disruption of the BBB (Säljö et al., 2008; Long et al., 2009; Abdul-Muneer et al., 2013; Yeoh et al., 2013). The goal of this review is to confer studies that examined changes in the brain milieu causing BBB dysfunction or disruption and neuroinflammation following exposure to different intensities of BSWs. Moreover, potential of early intervention strategies capable of easing oxidative stress, repairing the BBB or blocking inflammation for diminishing delayed neurological deficits ensuing from exposure to BSWs is discussed.

## Blood-brain barrier—structure and function

**Figure 1:**
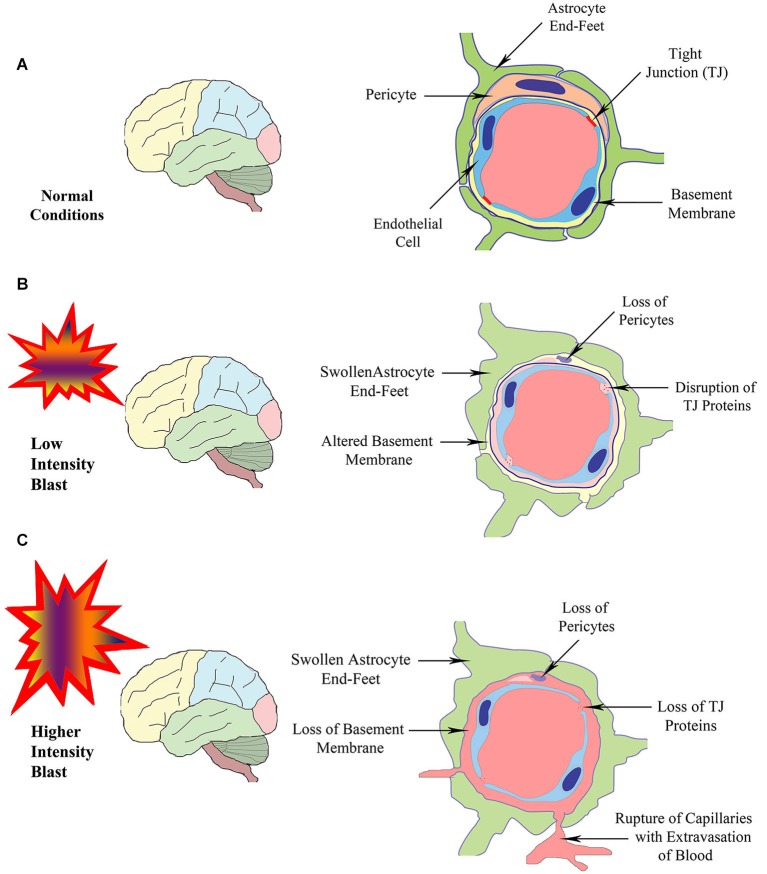
**Schematic illustrating the structure of the BBB in normal conditions (A), and following exposure to BSWs of lower intensity (B) or higher intensity (C)**. Exposure to lower intensity BSWs likely result in disruption of TJ proteins, damage or changes to the BM, detachment or loss of pericytes and swelling of astrocyte end-feet in brain microvessels **(B)**. In contrast, exposure to higher intensity BSWs likely cause direct rupture of brain microvessels causing the loss of TJ proteins, BM, pericytes and endothelial cells, swelling of astrocyte end-feet and extravasation of blood (hemorrhage) into perivascular areas **(C)**.

**Figure 2:**
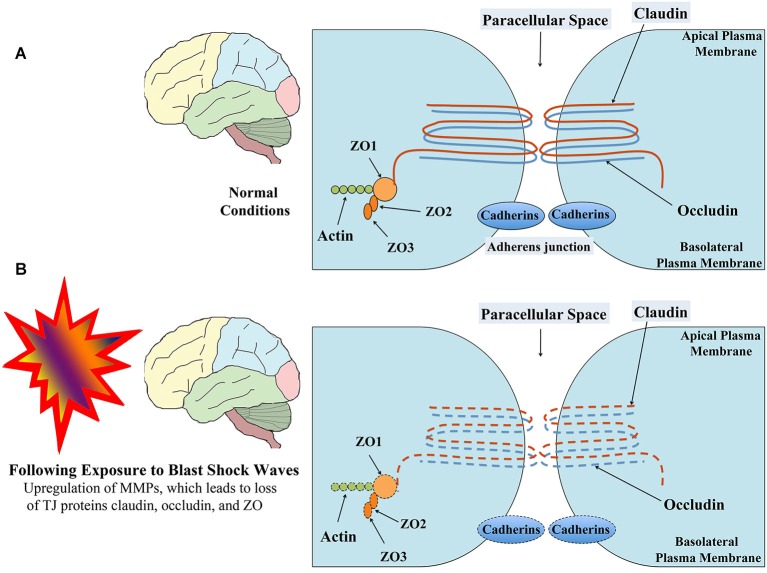
**Schematic showing the structure of TJ between brain endothelial cells in normal conditions (A) and following exposure to low-intensity BSWs (B)**. TJs mainly comprise trans-membrane proteins claudin and occuldin anchored to cytoplasmic proteins zona occludens-1 (ZO1), ZO2 and ZO3. Following exposure to shock waves, both trans-membrane and cytoplasmic proteins of TJ undergo modifications or loss resulting in leaky BBB. *Figure adapted from Ballabh et al. (2004) and Engelhardt and Sorokin (2009)*.

The BBB, a multicellular vascular structure, acts as a diffusion barrier to prevent the inflow of most compounds from blood to brain and thereby maintains brain homeostasis (Ballabh et al., 2004; Obermeier et al., 2013). The endothelial cells that form the walls of the capillaries are the primary components of the BBB in the mammalian brain and spinal cord (Figure 1A). The combined surface area of these capillaries forms the principal interface for blood-brain exchange (Abbott et al., 2010). Anatomically, the BBB is composed of: (i) brain endothelial cells; (ii) tight junctions (TJs) between endothelial cells; (iii) capillary basement membrane (BM); (iv) pericytes; and (v) astrocyte end-feet (Figure 1A). Contrasting to endothelial cells in the rest of the body, endothelial cells making up the BBB do not display fenestrations and undergo low rates of transcytosis. However, they have more extensive TJs, which are the locations of fusion linking the outer leaflets of plasma membrane of adjacent endothelial cells (Figure 2A).

The diffusion barrier is a consequence of the selectivity of TJs to impede the passage of most blood-borne substances from inflowing into the brain (Ballabh et al., 2004; Abbott et al., 2010). The TJs are composed of transmembrane proteins such as occludin, claudins, and junctional adhesion molecule (Figure 2A). All of these proteins are anchored into the endothelial cells by cytoplasmic protein complex comprising zonula occludens proteins 1–3 (ZO-1-3) and cingulin (Ballabh et al., 2004; Engelhardt and Sorokin, 2009). The TJs limit the flux of hydrophilic molecules across the BBB while smaller lipophilic substances such as O_2_ and CO_2_ diffuse freely across plasma membranes along their concentration gradient (Grieb et al., 1985). The other functions of the BBB include maintaining ionic compositions at optimal levels for synaptic signaling function via specific ion channels and transporters, keeping the pools of central and peripheral neurotransmitters separate from each other, preventing macromolecules from entering the brain and shielding the CNS from neurotoxic substances circulating in the blood (Bernacki et al., 2008; Abbott et al., 2010). Transporters mediate the entry of nutrients such as glucose and amino acids across the BBB whereas receptor-mediated endocytosis facilitates the uptake of larger molecules such as insulin, leptin, and iron transferrin (Pardridge et al., 1985; Zhang and Pardridge, 2001; Ballabh et al., 2004). Pericytes ensheath the abluminal surfaces of cerebral vessel walls (Figure 1A). They are important for angiogenesis, structural integrity and differentiation of endothelial cells and formations of TJs (Allt and Lawrenson, 2001; Bandopadhyay et al., 2001; Ballabh et al., 2004), as injury to pericytes can result in microaneurysms (Lindahl et al., 1997). Pericytes are also believed to have a unique synergistic relationship with brain endothelial cells in the regulation of capillary permeability through secretion of cytokines, chemokines, nitric oxide, matrix metalloproteinases (MMPs), and by means of capillary contraction (Hurtado-Alvarado et al., 2014).

On the other hand, perivascular astrocyte end-feet (also referred to as glia limitans) encircle the abluminal side of cerebral vessels (Figure 1A). They are highly specialized and polarized structures having orthogonal arrays of intramembranous particles consisting of the most abundant water channel aquaporin-4 (AQP-4; Obermeier et al., 2013). Astrocyte end-feet are necessary for the induction and maintenance of the TJ barrier (Rubin et al., 1991; Ballabh et al., 2004). Taken together, every constituent cell type makes a crucial contribution to the BBB’s integrity. When one member of the BBB fails, the barrier can break down and lead to dramatic consequences, and neuroinflammation and neurodegeneration can ensue.

## Mechanisms of BBB disruption in disease conditions

The barrier function of the BBB is not always rigid, as it undergoes modulation and regulation, both in physiology and pathology (Abbott et al., 2010). The barrier dysfunction can range from mild and transient TJ opening to chronic barrier breakdown (Förster, 2008). Chronic BBB dysfunction can exacerbate the overall brain pathology and contribute to persistent neurological deficits, as it leads to increased extravasation of immune cells, poorly regulated flux of molecules and ions, impaired transport processes (Abbott et al., 2010; Obermeier et al., 2013). Under normal situations, a small number of mononuclear leukocytes, monocytes and macrophages may enter the adult CNS through the cytoplasm of endothelial cells (via a process called diapedesis) (Engelhardt and Wolburg, 2004). This low-level leukocyte trafficking across the BBB is believed to be for immune surveillance and to effect responses to brain infections (Obermeier et al., 2013). However, in pathological conditions (such as trauma, ischemia, stroke, status epilepticus, multiple sclerosis etc.), these cells infiltrate in large numbers into the CNS and perform roles similar to the resident microglia such as debris clearing (Scholz et al., 2007; Davoust et al., 2008; Abbott et al., 2010).

Classically, sites of BBB inflammation attract circulating neutrophils and mononuclear cells. It is believed that pericytes control the migration of leukocytes in response to inflammatory mediators by upregulating the expression of adhesion molecules and releasing chemoattractants (Hurtado-Alvarado et al., 2014). Neutrophils and mononuclear cells penetrate the barrier and form cuffs in the perivascular space particularly around small vessels. It is believed that the perivascular space acts as a specific niche for synchronized immune reaction (Bechmann et al., 2001; Konsman et al., 2007; Abbott et al., 2010). A multitude of factors can disrupt the BBB, which include secreted elements to immune cells and pathogens, reactive oxygen species (ROS), activation of MMPs, and chronic up-regulation of angiogenic factors and pro-inflammatory cytokines. When BBB integrity is compromised, it may manifest initially as increased barrier permeability with reduced expression of TJ proteins. However, depending upon the severity, it may show other features such as pericyte detachment, astrocyte end-feet swelling or loss, and disrupted BM (Obermeier et al., 2013). Disruption of the BBB eventually culminates in neuronal dysfunction, neuroinflammation and neurodegeneration (Obermeier et al., 2013).

## Mechanisms of BBB disruption following exposure to blast induced shock waves

An *in vitro* study, utilizing a shock tube driven by compressed gas to generate operationally relevant, ideal pressure profiles consistent with improvised explosive devices, examined the effects of blast waves on the integrity of BBB function (Hue et al., 2013). Several measures demonstrated that barrier function of an in vitro BBB model gets disrupted with exposure to a range of controlled blast loading conditions. This was evidenced through: (i) an acute decrease in trans-endothelial electrical resistance (TEER) in a dose-dependent manner (which correlated with impulse rather than peak overpressure or duration); (ii) increased hydraulic conductivity and solute permeability post-injury across the barrier; and (iii) compromised ZO-1 immunoreactivity. This study provided the evidence that immediate disruption of BBB can occur with exposure to primary blast waves.

Studies using animal models have also reported the BBB dysfunction and disruption with exposure to BSWs. A recent study suggests that low intensity BSWs initially induce oxidative and nitrosative damage, which in turn causes BBB disruption and leads to cerebrovascular inflammation (Figure 1B; Abdul-Muneer et al., 2013). This study, using a 9-inch square cross-section shock tube and young rats, investigated the kinetic profile of one-time 123-kPa intensity blast exposure on the underlying mechanisms of cerebrovascular injury at 1, 6, 24 and 48 h post-exposure. They found the following changes in brain capillaries. First, there was considerable oxidative stress at 1–24 h after exposure to BSWs. This comprised induction of: (i) free-radical generating enzymes, NADPH oxidase 1 (NOX1) and inducible nitric oxide synthase (iNOS); (ii) oxidative/nitrosative damage markers such as 4-Hydroxynonenl (4-HNE, a major end product of lipid peroxidation) and 3-nitrotyrosine (3-NT, a product of tyrosine nitration mediated by ROS). These results imply that, single shock-wave exposure of 123-kPa intensity is sufficient to induce considerable oxidative/nitrosative stress in brain capillaries. Second, oxidative stress in capillaries progressed into BBB disruption (Figure 1B). This was evidenced through considerable loss of TJ proteins occludin, claudin-5, and zonula occluden 1 (ZO-1), and reduced expression of PDGFR-beta (a marker of pericytes) (Figure 2B). Third, oxidative stress also activated the expression of several matrix metalloproteinases (MMP2, MMP3, MMP9) capable of digesting TJs, BM proteins and degradation of perivascular units (Abdul-Muneer et al., 2013). Fourth, the expression of AQP-4 (a water channel protein typically associated with astrocyte end-feet at the BBB) was upregulated in perivascular regions as well as the cortical tissue causing edema around cerebral vessels. Fifth, adhesion and infiltration of macrophages were increased in the microvessels and plasma samples showed higher levels of S-100 beta protein (at 6 h post-exposure) and neuron-specific enolase (appeared at 6 h and continued beyond 24 h post-exposure). Sixth, significant numbers of endothelial cells expressed caspase-3 and some endothelial cells expressed TUNEL implying that they are undergoing apoptosis. Thus, exposure to even a relatively low intensity BSWs can cause BBB disruption, inflammation and neurodegeneration.

In agreement with the above findings, another rat study using a blast overpressure of 129 kPa (Cho et al., 2013) showed increased ROS generation in the brain as early as 4 h and persistence of upregulated ROS until 2 weeks post-exposure. This study also found enhanced expression of genes encoding inflammation (interferon gamma [IFNγ] and monocyte chemoattractant protein-1 [MCP-1]) at 4 h and IFNγ and MCP-1 proteins at 24 h post-exposure. Additionally, animals displayed memory impairment in a novel object recognition test and increased density of Iba-1+ activated microglia in brain regions at 2 weeks post-exposure.

Several other studies have examined the effects of higher intensity shock waves. Yeoh et al. (2013) quantified cerebrovascular injury in rats exposed to moderate to intense BSWs (145, 232 and 323 kPa), using a rifle primer-driven shock tube. Cerebrovascular injury was quantified via measurements of the areas of extravasation of immunoglobulin G (IgG) around brain capillaries. They found small lesions (i.e., areas of IgG extravasation) scattered throughout the brain. It was also observed that both size and number of lesions increased with peak overpressure level (Yeoh et al., 2013). Red blood cells were associated with some extravasations implying some minor hemorrhage or coagulation of blood within capillaries. However, no significant difference was seen between acute and 48 h survival times, implying that all vascular lesions are acute and represent primary effects of the exposure to shock waves rather than delayed BBB opening associated with inflammation (Yeoh et al., 2013). Thus, exposure to high intensity shock waves causes acute cerebrovascular injury resulting in immediate BBB disruption, evidenced through extravasation of IgG and hemorrhage around capillaries (Figure 1C).

Another study by Tompkins et al. (2013) using a rat model and a blast overpressure of 80 psi (equivalent to ~552 kPa) reports that polymorphonuclear leukocytes and lymphocytes infiltrate the brain parenchyma within an hour after exposure to BSWs. Furthermore, cells (neurons/glia) immunoreactive for cyclo-oxygenase-2 (COX-2, an inflammatory mediator involved in the cyclo-oxygenase pathway), interleukin-1 beta and tumor necrosis factor-alpha (pro-inflammatory cytokines) and 4-HNE (a marker of lipid peroxidation) could be seen as early as an hour after the exposure to BSWs. Most of these cells persisted for at least 3 weeks after the exposure. Cells immunoreactive for cleaved caspase-3 were also seen 3 weeks after the exposure. Additionally, magnetic resonance imaging showed hyper-intense regions in the somatosensory area within an hour after the exposure. The animals exposed to BSWs also exhibited hippocampus-dependent cognitive dysfunction at 5–12 days and axonal damage at 3 weeks post-exposure.

Taken together, it appears that exposure to relatively lower intensity BSWs causes oxidative stress and MMP activation in brain capillaries, which then evolves into disruption of the BBB and vascular edema formation with apoptosis of ECs and leads to inflammation with infiltration of leucocytes and macrophages. In support of these findings, an earlier study using a mouse model has shown specific neurodegeneration developing in perivascular areas following exposure to BSWs (Goldstein et al., 2012). Thus, in situations involving exposure to low-intensity shock waves, oxidative stress precedes the BBB disruption and/or inflammation (Abdul-Muneer et al., 2013; Cho et al., 2013). However, in circumstances where exposures to higher intensity shock waves occur, disruption of the BBB appears to occur swiftly through acute rupture of cerebral blood vessels, which is immediately followed by increased oxidative stress and early inflammation (Tompkins et al., 2013; Yeoh et al., 2013). Thus, BBB disruption appears to be the major primary injury that leads to neuroinflammation and neurodegeneration following exposure to milder to more intense BSWs.

## Links between shock-wave induced early changes such as BBB disruption and delayed neurological disorders

mTBI incurred through single or repeated exposure to BSWs can lead to a variety of neurological problems at months or years after the incident. The symptoms may include headache, sensitivity to light and noise, behavioral changes, attention and memory deficits, loss of problem solving abilities, anxiety, post-traumatic stress disorder (PTSD), and post-traumatic epilepsy (Trudeau et al., 1998; Hicks et al., 2010; Rosenfeld and Ford, 2010; Bogdanova and Verfaellie, 2012; Tomkins et al., 2013). Furthermore, individuals with mTBI appear to have increased mental stress or depression and are likely to display higher tendency for alcohol misuse and/or drug abuse (Wilk et al., 2010; MacDonald et al., 2014). Based on animal model studies, early changes after exposure to BSWs mainly include increased intracranial pressure (Leonardi et al., 2011), deformation of brain areas in some cases (Bayly et al., 2006), considerable oxidative stress in brain capillaries and BBB disruption. This is typically followed by neuroinflammation typified by the appearance of activated microglia and increased concentration of pro-inflammatory cytokines and neurodegeneration in perivascular regions (Kaur et al., 1995, 1996, 1997; Abdul-Muneer et al., 2013), and atypical distribution of phosphorylated neurofilaments in neurons (Säljö et al., 2000). Furthermore, a mouse study has reported that multifocal axonal injury in white matter tracts typically occurs only when the torso of animal is not shielded during the exposure to shock waves (Koliatsos et al., 2011). However, a diffusion tensor imaging (DTI) study in rats with chest protection noted significant interactions in axial and radial diffusivity in a number of subcortical structures at 2 h after exposure to BSWs but not at 42 days post-exposure (Kamnaksh et al., 2014). Likewise, another investigation found that some of the behavioral abnormalities (depression, anxiety) observed a day after the exposure to multiple shock waves spontaneously recovered by 16 days post-exposure although the histology showed apoptotic cells as early as 2 h after exposure in the dorsal and ventral hippocampus and persistence of apoptotic cells in the ventral hippocampus until 22 days post-exposure (Kamnaksh et al., 2012). A recent study, in addition, demonstrates that the hippocampus is vulnerable to high (165 kPa) as well as low (69–97 kPa) intensity BSWs, based on the occurrence of one or more signs of neurodegeneration such as activation of cleaved caspase-3 and loss of neurons, activation of microglia and hypertrophy of astrocytes at 7 days post-injury (Sajja et al., 2014). Interestingly, this study also revealed increased expression of genes encoding neurotrophic factors and antioxidants in the hippocampus following exposure to BSWs, implying that a healthy brain attempts to self-repair or minimize adverse alterations through activation of innate neuroprotective mechanisms.

Long-term structural changes resulting from exposure to BSWs include diffuse axonal degeneration in white matter tracts and inflammation. A study by Goldstein et al. (2012) demonstrated chronic traumatic encephalopathy (CTE) in postmortem brains of US military veterans exposed to blasts. Chronic traumatic encephalopathy, a tau protein-linked progressive neurodegenerative disease associated with memory loss, impaired judgment and depression, is typically seen in people who undergo repetitive brain concussions. However, emergence of CTE may take months, years or even decades after the last concussion (Maroon et al., 2014). Interestingly, Goldstein et al. (2012) found changes similar to CTE in a mouse model 2 weeks after exposure to BSWs, which comprised phosphorylated tauopathy, myelinated axonopathy, microvasculopathy, chronic neuroinflammation and neurodegeneration in the absence of macroscopic tissue damage or hemorrhage. Behavioral studies demonstrated hippocampus-dependent learning and memory deficits for a month after the exposure, which correlated with impairments in axonal conduction and activity-dependent long-term potentiation of synaptic transmission (Goldstein et al., 2012). Moreover, head immobilization during blast exposure prevented blast-induced learning and memory deficits, implying that head acceleration caused by BSWs plays a major role in inducing brain pathology and cognitive impairments.

Furthermore, a recent behavioral study at an extended time-point (6 months) after the exposure to BSWs using a mouse model suggests that single exposure of moderate BSWs to head is adequate for developing chronic cognitive and mood dysfunction (Mishra et al., 2014). Cognitive impairments were seen for hippocampus-dependent spatial memory retrieval function in a water maze test, perirhinal cortex-dependent object recognition function in a novel object recognition test, and dentate gyrus dependent pattern separation function (i.e., ability to discern minor changes in the environment) in a pattern separation test. Mood impairments were evidenced through novelty suppressed feeding and forced swim tests (Mishra et al., 2014). Analyses of the hippocampus at 8 months after the exposure revealed considerably decreased neurogenesis, a substrate important for maintenance of hippocampus-dependent cognitive function (particularly for pattern separation function) and mood (Shetty et al., 2014). Additionally, DTI of fixed brains *ex vivo* revealed significant white matter (corpus callosum) alterations in a subset of animals, which are typified by increased radial diffusivity (a marker of myelin degradation) and decreased relative anisotropy (implying asymmetry of water mobility) (Shetty et al., 2014). Thus, exposure to BSWs can dampen hippocampus neurogenesis and initiate progressive myelin degradation in white matter tracts on a long-term basis. These changes likely contribute to cognitive and mood dysfunction observed in people exposed to BSWs.

From the above perspectives, it appears that multiple acute changes observed in the brain after exposure to BSWs (such as oxidative stress, BBB disruption, neuroinflammation, diffuse axonal injury and sporadic neurodegeneration) initiate lasting pathological cascades that eventually evolve into persistent neurological dysfunction typified by cognitive and mood impairments (Terrio et al., 2009; Elder et al., 2010; Gavett et al., 2010; Cho et al., 2013). Nonetheless, it is currently unclear whether these early changes trigger long-standing secondary changes (such as alterations in structure and function of neurons and synapses, neuron-glia communication and neurochemistry, diminished hippocampus neurogenesis and axonal degeneration) or just persist at lower levels for prolonged periods and interfere with the normal brain function. First, it remains to be determined whether the repair of BBB occurs completely over days/weeks after exposure to BSWs or remains somewhat leaky or dysfunctional for protracted periods after exposure because of alterations in astrocyte end-feet, loss of pericytes and malfunction of TJs. Second, it is unclear whether increased oxidative stress seen in perivascular regions and in some cases within brain parenchyma persists at variable levels for prolonged periods. For example, decreased hippocampus neurogenesis observed at 8 months post-exposure (Shetty et al., 2014) could be because of oxidative stress induced loss of neural stem/progenitor cells in the early period after exposure and/or lingering oxidative stress and inflammation impairing neural stem/progenitor cell activity in the hippocampus. Proteomic analyses of plasma from blast injured animals however showed some indirect evidence regarding the persistence of oxidative stress triggered by hypoxia (based on markers 4-HNE, hypoxia-inducible factor-1α and ceruloplasmin) and vascular abnormalities such as BBB leakiness (based on the presence of von Willebrand Factor) at 42 days post-exposure (Ahmed et al., 2013). Thus, correlative histological, biochemical and behavioral studies at multiple time-points after exposure to BSWs are needed in the future. These studies may help in determining whether early pathological changes (oxidative stress, BBB disruption and neuroinflammation and axonal injury): (i) trigger secondary pathological changes in neurons and glia; (ii) impair the function of neural stem/progenitor cells to decrease net hippocampus neurogenesis; (iii) persist for prolonged periods; and (iv) progressively expand to involve more regions of the brain.

## Can mitigation of shock wave induced early changes (BBB disruption and neuroinflammation) restrain delayed neurological complications?

Neuroprotective drugs capable of halting or mitigating secondary changes following blast shock wave induced oxidative stress and/or BBB disruption may considerably ease or slow down the development of subsequent neurological and neuropsychiatric impairments such as cognitive problems, memory dysfunction, non-specific mental and emotional symptoms, chronic motor deficits and PTSD (Chen et al., 2013). While the clinical management of blast related brain injury comprises treatment for reducing cerebral edema, intracranial hemorrhage and cerebral vasospasm (Chen et al., 2013), an efficient neuroprotective drug therapy capable of blocking secondary neurological complications of blast-related brain injury is yet to be identified. Hitherto, efficacy of only a few neuroprotective drugs has been assessed in animal models of blast injury. Kovesdi et al. (2012) investigated whether acute treatment with the non-steroidal anti-inflammatory drug minocycline can mitigate the neurobehavioral abnormalities resulting from exposure to BSWs. In this study, 4 h after a single exposure to mild blast overpressure, animals received minocycline (at 50 mg/Kg, once daily for 4 days). Interestingly, memory and anxiety analyses performed at 8 and 45 days post-exposure revealed that blast exposed animals receiving minocycline have similar memory and anxiety scores as control animals whereas blast exposed animals receiving vehicle displayed memory dysfunction and increased anxiety. Furthermore, minocycline treatment normalized serum and tissue levels of several selected inflammatory, vascular, neuronal, and glial markers, implying that blockage of inflammation cascade occurring early after blast-induced BBB damage has promise for easing blast shock wave exposure induced cognitive and mood dysfunction (Kovesdi et al., 2012).

**Figure 3:**
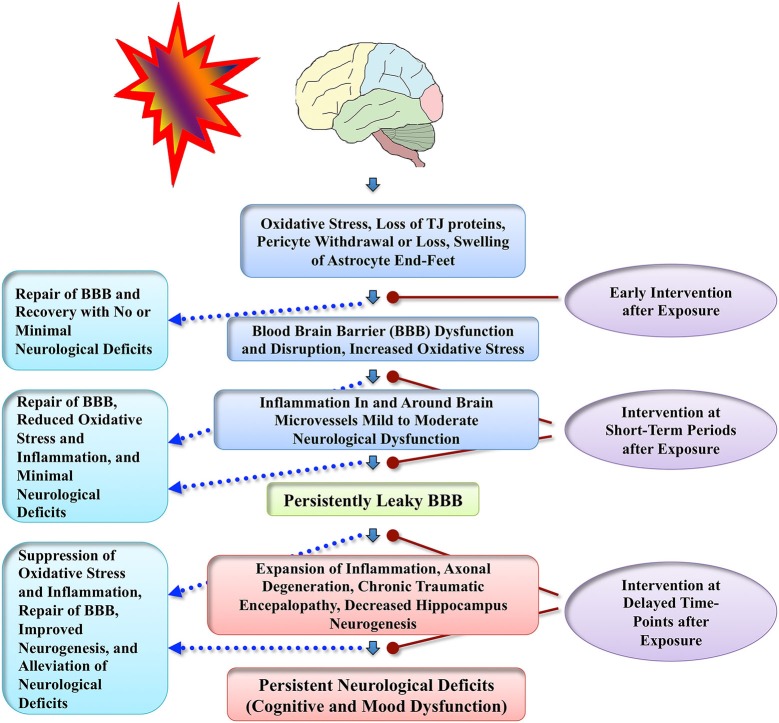
**Schematic illustrates conceived sequence of events that precede persistent neurological deficits following exposure to BSWs**. Adverse changes likely commence with increased oxidative stress causing dysfunction or disruption of the BBB and inflammation around brain microvessels, which then, depending on the extent of initial injury, evolve into multiple chronic changes such as persistently leaky BBB, expansion of neuroinflammation, axonal degeneration, CTE, and decreased hippocampus neurogenesis. These chronic changes likely underlie neurological impairments seen in patients with mTBI resulting from exposure to BSWs. Figure also illustrates the possible benefits of apt neuroprotective interventions applied at different time-points after the exposure to BSWs. Efficient early neuroprotective interventions (hours after exposure) may repair the BBB quickly and facilitate both structural and functional recovery with no or minimal neurological deficits. Interim interventions (days or weeks after exposure) may be useful for suppressing oxidative stress and inflammation, which would likely repair the BBB and prevent the evolution of initial injury into long-term neurological deficits. Delayed interventions (months or years after exposure) may suppress the chronic oxidative stress, inflammation and axonal degeneration and thereby alleviate neurological deficits that are already present.

Du et al. (2013) examined the efficacy of antioxidant treatment for blast-related brain injury in a rat model. Rats received a combination of antioxidants (2,4-disulfonyl α-phenyl tertiary butyl nitrone and N-acetylcysteine), an hour after exposure to 14 psi blast overpressure and then twice a day for the following 2 days. Antioxidant treatment reduced 4-HNE, amyloid precursor protein (APP) and neurofilament 68 (NF-68) expression in the hippocampus, 4-HNE expression in the corpus callosum, c-fos expression in the retrosplenial cortex, APP and NF-68 expression in the auditory cortex and medial geniculate nucleus. Although the effects of antioxidants on blast shock wave induced long-term behavioral abnormalities were not examined in this study, the results suggest that antioxidant therapy has promise to ease neurological deficits associated with blast-related brain injuries. Another recent study using a rabbit model suggests that hyperbaric oxygen therapy starting 12 h after exposure to BSWs is neuroprotective, based on observations such as maintenance of the BBB integrity, and inhibition of brain edema, apoptosis and inflammation (Zhang et al., 2014). Overall, neuroprotective studies in blast-induced brain injury models are still in nascent stages partly because pathophysiological sequences of blast-related brain injury are still being worked out in animal models of distinct blast-related brain injuries. However, we will likely see multiple neuroprotective studies in blast-related brain injury models in the coming years, as the number of neuroscientists working on this field has been growing considerably over the last few years. A schematic in Figure 3 illustrates the possible outcome of interventions applied at different time-points after exposure to BSWs.

## Conclusions

Aspects such as activation of MMPs, and increased levels of ROS and pro-inflammatory cytokines in brain capillaries observed after exposure to BSWs can disrupt the BBB function. Even relatively moderate alterations in the BBB function, such as reduced expression of TJ proteins observed after exposure to milder intensity shock waves, can increase barrier permeability and cause moderate levels of inflammation (e.g., perivascular cuffing) and neuronal dysfunction. On the other hand, a major disruption of the BBB with the loss of pericytes, considerable swelling of astrocyte end-feet and disruption of the BM expected after exposure to moderate to higher intensity of BSWs can cause more robust and long-lasting neuroinflammation and lead to substantial neurodegeneration. If spontaneous repair of the BBB does not occur over days or weeks after such injuries, persistently leaky BBB can contribute to expansion of neuroinflammation, neuronal dysfunction and neurodegeneration in larger areas of the brain. Such changes may cause persistent neurological impairments such as cognitive problems, memory dysfunction, sleep disorder, depression and anxiety, depending upon the regions of brain afflicted with such changes.

However, it remains to be determined whether early changes in cerebral vessels observed after exposure to BSWs trigger long-standing secondary changes or just persist at lower levels for prolonged periods and interfere with the normal brain function. Therefore, to understand BSW induced sequential pathological changes occurring in the brain over days to months, multidisciplinary studies that not only assess structural and neurochemical changes in the brain but also the associated behavioral deficits and alterations in electrical and synaptic properties of neurons will be needed in animal models at multiple early and extended time-points after exposure to BSWs. While animal models may not exhibit all features of blast-related brain injury occurring in humans, they are nonetheless useful for unraveling sequential pathological changes that occur in the brain following an exposure to different intensities of BSWs. Therefore, rigorously examining compounds and drugs that have shown ability to repair the BBB injury, suppress neuroinflammation and provide neuroprotection in other disease models, using well established and reproducible blast-related brain injury models would be beneficial to determine whether an early intervention therapy after exposure to BSWs can block or at least restrain the development of delayed neurological deficits.

## Conflict of interest statement

The authors declare that the research was conducted in the absence of any commercial or financial relationships that could be construed as a potential conflict of interest.
