# Corrigendum: Blood brain barrier dysfunction and delayed neurological deficits in mild traumatic brain injury induced by blast shock waves

**DOI:** 10.3389/fncel.2014.00404

**Published:** 2014-11-26

**Authors:** Ashok K. Shetty, Vikas Mishra, Maheedhar Kodali, Bharathi Hattiangady

**Affiliations:** ^1^Institute for Regenerative Medicine, Texas A&M Health Science Center College of Medicine at Scott & WhiteTemple, TX, USA; ^2^Department of Molecular and Cellular Medicine, Texas A&M Health Science Center College of MedicineCollege Station, TX, USA; ^3^Research Service, Olin E. Teague Veterans Affairs Medical Center, Central Texas Veterans Health Care SystemTemple, TX, USA

**Keywords:** blast-related brain injury, blast shock waves, blood-brain barrier leakage, chronic traumatic encephalopathy, mild traumatic brain injury, neuroinflammation, oxidative stress, vascular resistance

**The following reference in “References” section:**

Goldstein, N., Goldstein, R., Terterov, D., Kamensky, A. A., Kovalev, G. I., Zolotarev, Y. A., et al. (2012). Blood-brain barrier unlocked. *Biochemistry (Mosc.)* 77, 419–424. doi: 10.1134/s000629791205001X

**Should be replaced by:**

Goldstein, L. E., Fisher, A. M., Tagge, C. A., Zhang, X. L., Velisek, L., Sullivan, J. A., et al. (2012). Chronic traumatic encephalopathy in blast-exposed military veterans and a blast neurotrauma mouse model. *Sci. Transl. Med.* 4:134ra60. doi: 10.1126/scitranslmed.3003716

## Conflict of interest statement

The authors declare that the research was conducted in the absence of any commercial or financial relationships that could be construed as a potential conflict of interest.

